# Biological Function of Plant Tannin and Its Application in Animal Health

**DOI:** 10.3389/fvets.2021.803657

**Published:** 2022-01-10

**Authors:** Zhenkai Tong, Wenfeng He, Xiao Fan, Aiwei Guo

**Affiliations:** Faculty of Life Sciences, Southwest Forestry University, Kunming, China

**Keywords:** plant tannins, classification, biological function, application, extraction source

## Abstract

Plant tannins are widely found in plants and can be divided into hydrolyzed tannins and condensed tannins. In recent years, researchers have become more and more interested in using tannin-rich plants and plant extracts in ruminant diets to improve the quality of animal products. Some research results show that plant tannins can effectively improve the quality of meat and milk, and enhance the oxidative stability of the product. In this paper, the classification and extraction sources of plant tannins are reviewed, as well as the biological functions of plant tannins in animals. The antioxidant function of plant tannins is discussed, and the influence of their structure on antioxidation is analyzed. The effects of plant tannins against pathogenic bacteria and the mechanism of action are discussed, and the relationship between antibacterial action and antioxidant action is analyzed. The inhibitory effect of plant tannins on many kinds of pathogenic viruses and their action pathways are discussed, as are the antiparasitic properties of plant tannins. The anti-inflammatory action of tannins and its mechanism are analyzed. The function of plant tannins in antidiarrheal action and its influencing factors are discussed. In addition, the effects of plant tannins as feed additives on animals and the influencing factors are reviewed in this paper to provide a reference for further research.

## Introduction

Plant tannins are polyphenols that are widely found in terrestrial plants and in some marine plants (phloroglucinol). Plant tannins have been used as additives in animal production for many years (1). They may affect metabolism or the gut microbiota (2, 3), with the aim of improving performance or meat quality (4). Patra and Saxena (5) showed that plant tannins can improve feed efficiency and animal health. Aboagye et al. (6) found that plant tannins have a strong affinity for protein, and the appropriate addition of plant tannins to ruminant has nutritional value. In contrast, it is believed that the addition of a high concentration of plant tannins to the diets of single-stomached animals will cause adverse effects such as reduced nutrient availability and performance of animals (7, 8). Schiavone et al. (9) also demonstrated that adding 0.20% plant tannins to the broiler diet can increase feed intake and average daily gain, while 0.25% tannins has a negative impact on broiler performance. It can be concluded that plant tannins have enormous potential as feed additives (10–12). However, plant tannins are complex substances whose beneficial and harmful properties depend on their chemical structure, the concentration and other factors. Therefore, this paper reviewed the classification, extraction sources and biological functions of plant tannins, as well as the factors influencing the additive effect, providing a reference for further research.

## Classification and Sources of Plant Tannins

### Classification of Plant Tannins

The chemical structures of plant tannins are diverse, and systematic classification of tannins based on specific structural characteristics and chemical properties can provide a convenient framework for related research. Plant tannins can be broadly divided into hydrolyzed tannins and condensed tannins. Hydrolyzed tannins consist of polyphenol nuclei with molecular weights ranging from 500 to 3,000 Daltons (Da) (13). Condensed tannins are oligomeric or polymeric flavonoids composed of flavane-3-ols, including catechin, epicatechin, gallocatechin, and epigallocatechin. Their molecular weights vary from 1,000 to 20,000 Da, they depolymerize only with strong oxidation and acid, and they are not easily degraded by anaerobic enzymes (14).

Plant tannins can be broadly divided into two categories, which are relatively general. There are ellagic tannins and polyol residues that cannot be completely hydrolyzed in hydrolyzed tannins (15), but they are divided into hydrolyzed tannins. Hydrolyzed tannins were first described as containing the C-glycoside catechin in addition to the characteristic structure of monomer ellagins in 1985 (16). At first, it was unscientific to classify these plant tannins into hydrolyzed tannins because C-C-coupled catechin structures contained some glycosides, and only some structures could be hydrolyzed (17). Therefore, tannins were further classified into four major categories according to their chemical structure: gallotannins refer to the combination of galloyl groups or their derivatives with polyols, catechins or triterpenoids; ellagitannins refer to at least two gallic C-Cs conjugated to each other and catechins conjugated without glycoside; compound tannins refer to glucogenated catechins in addition to gallic tannins or ellagic tannins and condensed tannins mean that C-4 of catechin is linked to C-8 or C-6 of another catechin unit to form oligomer procyanidins and polymeric procyanidins (18).

### Extraction Sources of Plant Tannin

Plant tannins are widely distributed in the plant kingdom, especially in herbages, shrubs, cereals and medicinal materials (19). They are also found in many fruits, such as bananas, blackberries, apples and grapes (20–23). Complex tannins and condensed tannins are the most common and easy to extract from legumes, trees and shrubs; Gallic tannins are commonly found in gallnuts, lacquer leaves and cotinus leaves, while ellagic tannins are commonly found in oaks, blackberries and pomegranates. Plant tannins are more abundant in vulnerable parts of plants, such as new leaves and flowers (24). Because the chemical structure and content of plant tannins vary greatly among different plant species, growth stages and growth conditions (such as temperature, light, and nutrients), the biological functions of different extraction sources vary (25, 26).

## Biological Functions and Influencing Factors of Plant Tannins

### Antioxidant Activity

The antioxidant properties of tannins are widely utilized in the food and medical fields. In recent years, many studies have been conducted to identify the relevant antioxidant activity of tannins. Owing to its antioxidant capacity, such as preventing cardiovascular disease, cancer or osteoporosis, tannins have attracted much attention (27, 28). In a study by Phung et al., the extracts of Japanese chestnut exhibited the most remarkable DPPH-scavenging capacity. The results suggested that as a potential natural preservative agent, tannins provided promising antioxidant capacities (29). A study determined the effects of the forage conservation method and condensed tannins (CT) from conserved forage on rumen fermentation and showed that CT from purple prairie clover (PPC) decreased protein degradation *in vitro* but had minimal effects on overall rumen fermentation (30). CT from the leaves of *F. altissima* was also indicated to express superior antioxidant capacity (31).

At present, plant tannins have been proven to have antioxidant properties in different animals. A study in mutton showed that the color stability of the longissimus dorsi muscle (LM) was extended by tannin supplementation, with lower changes in the hue angle in the treatment groups than in the control groups (32). In Rex rabbits, adding tannic acid to the diet significantly increased the activity of serum total superoxide dismutase (T-SOD) and decreased the malondialdehyde (MDA) content (33). Similar results were also demonstrated in broiler chickens. Grape seed extracts (GSEs), which contain tannins, significantly decreased serum total cholesterol, low-density lipoprotein cholesterol and meat malondialdehyde levels. GSEs also increased the antibody titer against the Newcastle disease virus vaccine (34).

To date, the mechanism of tannin antioxidant activity may remain unclear about the exact underlying mechanism involved. Some researchers have argued that the higher the relative molecular weight is, the stronger the antioxidant activity of tannins (35). The ability to scavenge free radicals depends on the number and polymerization degree of hydroxyl groups. The more hydroxyl groups in plant tannins, the more easily they can be oxidized; thus, tannins have higher antioxidant activity (36, 37). Gallic tannins are easily degraded in the gastrointestinal tract, while condensed tannins are difficult to degrade and absorb in the gastrointestinal tract. Therefore, it is difficult to explain the antioxidant performance of plant tannins in animals as a whole. The quebracho tannins were not digested and absorbed in the gastrointestinal tract but increased the antioxidant capacity of sheep liver and plasma, indicating that condensed tannins may indirectly affect the antioxidant function of animals (38). Some researchers also believe that condensed tannins form a protective film in the stomach by complexing with other molecules to protect the gastrointestinal tract and its contents from oxidation (39). Literature reviewed so far tends to demonstrate that the antioxidant function of plant tannins are mainly linked to their chemical structure rather than the extraction source (plant spp.). In addition, the specific antioxidant mechanism of plant tannins in animal tissues has not completely clarified, and further research is needed.

### Antibacterial Activity

As natural polyphenolic compounds, tannins display antibacterial effects. Research has investigated the effects of different levels of tannins on growth performance, intestinal microorganisms and morphology in piglets. A study found that tannins at 0.13, 2.25, and 0.45% significantly improved the feed conversion rate, reduced the concentrations of ammonia, isobutyric, and isovaleric acid in the cecum, decreased the depth of ileal crypts and reduced intestinal bacterial proteolysis (40). Studies showed that compared to the control, 250 or 500 mg/kg sweet chestnut tannin had no obvious effects on body weight and feed conversion in 41-day-old chickens. However, in 28-day-old chickens, 1,000 mg/kg tannins reduced the numbers of *E. coli* and other harmful bacteria in the small intestine. It has also been observed that tannins change the microorganism population quantity in the small intestine and colon in chickens (41). An experiment based on broilers investigated the effect of different concentrations of phenolic compounds from grape pomace (GP). The work showed that grape pomace might delay meat lipid oxidation, which was linked to an increase in the meat polyunsaturated fatty acid (PUFA) concentration (42). A hydrolyzable tannin extracted from chestnut was tested for efficacy in regulating the proliferation of *Clostridium perfringens* in the gut. The results showed that even at low concentrations (1.5–3.0 g/kg), chestnut tannins had a remarkable effect on controlling necrotic enteritis (NE). Compared to the control group, proliferation of *Clostridium perfringens* and gut damage were reduced by tannins in the treatment groups (11). Experimental studies on pigs demonstrated the effects of two compounds containing tannins. The gastrointestinal absorption of mycotoxins was reduced by grape pomace, which would be considered an alternative to commercial products. Gold grape seed extract (GSE) caused an ecological shift in the microbiome, significantly increasing the numbers of *Lachnospiraceae, Clostridales, Lactobacillus*, and *Ruminococcaceae*. This might be due to the bacterial populations or the structures of the compounds in the colon (43, 44). Based on the above results, tannins represent a potential alternative strategy to antibiotics in animal production. However, suppression of intracellular bacteria is also important (45) and needs to be further studied in plant tannins. Further investigation is necessary to determine the effects of tannins on bacterial conditions *in vitro* (46).

A bacteriostatic model was used to study the mechanism of tannins. Condensed tannins (CT) from purple prairie clover (*Dalea purpurea* Vent; PPC) were screened for anti-*Escherichia coli* O157:H7 activity against *E. coli* O157:H7 strain 3,081. After 24 h, optical density was measured to assess the growth of the bacteria at 600 nm. CT increased the lag time and reduced the growth rate of *E. coli* O157:H7. At the same time, CT decreased the proportions of unsaturated fatty acids in the total fatty acids and disrupted the outer membrane structure. The results showed that the possible mechanism was associated with fatty acid composition and the outer membrane of the cell (47). Synthesizing each index showed that tannins exert bacteria by inhibiting extracellular microbial enzymes and oxidative phosphorylation, which directly affects microbial metabolism, depriving microorganisms of substrates needed for growth and increasing membrane permeability (48).

It has been reported that the amount of hydroxyl groups in tannins and the release of hydrogen peroxide are important indicators to evaluate the antibacterial properties, which are positively correlated with antioxidant properties. For instance, trihydroxy b-cycloflavonol (gallic catechin) has stronger effects on *Streptococcus, Clostridium*, and *Staphylococcus* than dihydroxy b-cyclocatechin (43, 49). Because tannins come from a wide range of sources and have diverse antibacterial effects, screening and identifying tannins that are effective and specific to target microorganisms will be the work of ongoing research.

### Antiviral Activity

Some papers have reported that tannins have the ability to prevent viral infection such as HIV, bovine adeno-associated virus (BAAV) and norovirus. An herb screening model based on saliva-based binding/blocking assays using the NoV P protein was developed, and tannic acid was identified as a strong inhibitor in the binding of NoV to HBGA receptors. The data suggested that tannic acid (TA) is a promising antiviral agent against NoVs (50). Another study on TA and BAAV found that treatment with TA reduced BAAV transcytosis and increased lung transduction. The sorting and activity of the BAAV-expressed cystic fibrosis transmembrane regulator membrane protein were not impaired by TA (51). The inhibitory activity of tannins on HIV-1 was tested, and the results indicated that tannins inhibited syncytia formation, lytic effects, viral p24 antigen production and the activity of the HIV-1 reverse transcriptase (RT) enzyme (52). Another study on HIV-I integrase activity showed that some herb extracts display strong inhibition of integrase activity. The effect was most likely due to tannins in herb extracts (53). According to data from these studies, tannins are an effective compound against HIV-1 with high potential for further studies (54). In addition to the above viruses, tannins also inhibited other types of viruses, such as enterovirus. Research in mice demonstrated that chebulagic acid, as a hydrolyzable tannin, reduced the viral cytopathic effect on rhabdomyosarcoma cells. By inhibiting viral replication, chebulagic acid may reduce the mortality induced by a lethal dose of enterovirus 71 and relieve clinical symptoms (55). The beneficial effect was also reflected in the inhibition of hepatitis virus. *In vitro*, the results showed that (2)-epigallocatechin-3-gallate (EGCG) from green tea acted directly on hepatitis C virus (HCV) and prevented the virus from entering the cell surface (56). The same results were obtained in Huh7.5 cells, in which tannic acid, a polymer of gallic acid and glucose molecules, blocked cell-to-cell spread in infectious HCV cell cultures but did not inhibit HCV replication after infection (57). Hepatitis B virus (HBV) infection remains a major global health problem. In this study, hydrolyzable tannins such as punicalagin, punicalin, and geraniin reduced the production of HBeAg and the accumulation of cccDNA in HepDES19 and HepG2.117 cells. These hydrolyzable tannins may serve as new agents or strategies to block HBV infection (58). A study investigated the antiviral effects of tannins on 12 different viruses (enveloped and non-enveloped viruses). Extracts from persimmon showed strong antiviral effects against various viruses. Other tannins derived from green tea, acacia and gallnuts were effective against some viruses. Tannins interact with virion proteins, and restricted virus adsorption to the cells may be the possible mechanism of the inhibitory effect of tannins (59).

In summary, the antiviral function of tannins is realized through inhibiting the invasion of cells and nuclei by viruses, the activity of virus reverse transcriptase, and the transmission to other cells and promoting viral protein denaturation. However, due to their different sources and chemical structures, tannins have different antiviral properties.

### Antiparasitic Activity

Parasitic infections represent a major pathological threat to livestock and poultry production. Tannins represent a valuable option as an alternative to drugs for the control of parasites. *In vitro* bioassays evaluated the effects of condensed tannins (CTs) obtained from *Lotus pedunculatus* (LP), *Lotus corniculatus* (LC), *Dorycnium pentaphyllum* (DP), *Dorycnium rectum* (DR), and *Rumex obtusifolius* (RO) on egg hatching, larval development and the viability of *Teladorsagia circumcincta* (Stadelmann, 1894) (*Ostertagia circumcincta*) and the L3 larvae of *Trichostrongylus colubriformis* (Giles, 1892). These studies showed that CTs were able to inhibit egg hatching, slow larval development, kill undeveloped larvae and disrupt the life cycle of nematodes (60–62). Some experiments evaluated the anthelmintic effects of extracts from tropical tanniniferous plants (TTPs), such as *Acacia pennatula, Lysiloma latisiliquum, Piscidia piscipula* and *Leucaena leucocephala*, on *Haemonchus contortus*. These results suggested that tannins may be used as anthelmintics for gastrointestinal nematodes by interfering with the process of L (3) exsheathment (63, 64).

In addition, condensed tannins showed activity against free-living larvae and parasitic adults, confirming the potential role of tannins in the control of parasites at different growth phases (65, 66). In summary, the direct mechanisms involve restricting larval development to reduce the establishment of infected third-stage larvae in the host and decreasing spawning to inhibit the motion performance of the parasite, and the indirect mechanisms involve improving human immune function and resistance to infection. However, many factors influence the impact of tannins, such as plant sources of different varieties, the growth stage of parasites and the different host species.

### Anti-inflammatory Activity

Recently, some studies found that plant tannins have anti-inflammatory effects by inhibiting NO and prostaglandin-E2 (PGE2) (67). Most tannin extracted from different plants display anti-inflammatory functions. An *in vitro* assay using obese Zucker rats applied grape seed procyanidin extract to demonstrate that it can reduce obesity-induced inflammation by mediating the expression of cytokines (68). In addition, the tannin fraction of the extract from black raspberry seeds has anti-inflammatory activity to reduce nitric oxide (NO) induced by lipopolysaccharide (LPS) in RAW 264.7 cells (69). The anti-inflammatory function has also been demonstrated in croton oil-induced ear edema mice. In this study, hydrolyzable tannins from *Myricaria bracteata* showed a significant anti-inflammatory effect on mice (70). Tannins can form a gastroprotective barrier to improve gastritis symptoms based on their antioxidant activity (39).

Therefore, it is speculated that the anti-inflammatory properties of tannins from different sources may be caused by regulating cytokine expression, reducing the production of inflammatory substances and enhancing complexation with other molecules. However, the mechanism remains to be explored because animal experiments are still lacking.

### Antidiarrheal Activity

Plant tannins have shown antidiarrheal potential in animal models. A study performed by Bonelli et al. (71) shown that administration of tannins in calves with diarrhea may shorten the duration of the diarrheic episode (DDE). This research shows that some plant tannins have anti-diarrhea effects. In a mouse model of diarrhea induced by castor oil, Galla Chinensis oral solution (GOS) showed significant antidiarrheal activity, suggesting that GOS can be used to complement other therapies because it is an effective and stable antidiarrheal drug (72). In piglets, an experimental model for post-weaning diarrhea with enterotoxigenic *Escherichia coli* F4 (ETEC F4) was established, and then the effect of chestnut-tannin (1%) in preventing diarrhea was assessed. Tannins reduced the diarrhea rate and the duration of diarrhea (73). A study examined the effects of a hydrolyzable tannin extracted from Chinese gallnut (penta-m-digalloyl-glucose, PDG) on mouse diarrhea. The results showed that intraluminal injection of PDG reduced cholera toxin-induced intestinal fluid secretion (74). Overall, tannins are effective antidiarrhea compounds, and they may provide a new therapeutic intervention for diarrhea in animal production.

## Conclusion and Future Perspectives

As a natural polyphenolic substance, plant tannins have been found to have a variety of biological functions. The potential utility of plant tannins as feed additives is enormous in animal production. However, the addition of high tannins in animal diet needs to be very cautious.it may adversely affect the growth and development of animals and induce metabolic disorders, which may depend on the type and chemical structure of tannins, intake, dietary composition and animal species. In addition, different extraction sources and concentrations of plant tannins, as well as different animal species and physiological statuses, will influence the additive effect. At present, studies on the structure and nutritional characteristics of plant tannins are not sufficient. Most of the research has focused on complex plant tannins, while there have been few studies on the effects of single plant tannins. Meanwhile, cross-disciplinary studies are also insufficient, resulting in some conclusions still being limited to speculation. The mechanism of action is not yet clear. More studies should focus on the main active components, structural characteristics and mechanisms of plant tannins from different sources to ensure the accurate application of plant tannins in animal production. Further research is needed in the future.

## Author Contributions

ZT wrote this manuscript. WH and XF collected literature. AG reviewed the manuscript and given critical suggestions and comments. All authors read and approved the final manuscript.

## Funding

This work was supported by the Scientific and Technological Innovation Team Construction Project for Protection and Utilization of Under-Forest Biological Resources in Universities of Yunnan Province.

## Conflict of Interest

The authors declare that the research was conducted in the absence of any commercial or financial relationships that could be construed as a potential conflict of interest.

## Publisher's Note

All claims expressed in this article are solely those of the authors and do not necessarily represent those of their affiliated organizations, or those of the publisher, the editors and the reviewers. Any product that may be evaluated in this article, or claim that may be made by its manufacturer, is not guaranteed or endorsed by the publisher.
